# A sex-specific association of common variants of neuroligin genes (*NLGN3 *and *NLGN4X*) with autism spectrum disorders in a Chinese Han cohort

**DOI:** 10.1186/1744-9081-7-13

**Published:** 2011-05-14

**Authors:** Jindan Yu, Xue He, Dan Yao, Zhongyue Li, Hui Li, Zhengyan Zhao

**Affiliations:** 1 Department of Pediatric Health Care, Children's Hospital, Zhejiang University School of Medicine, Hangzhou, China; 2 Department of Gastroenterology, Children's Hospital, Zhejiang University School of Medicine, Hangzhou, China; 3 Department of Central Laboratory, Children's Hospital, Zhejiang University School of Medicine, Hangzhou, China

## Abstract

**Background:**

Synaptic genes, *NLGN3 *and *NLGN4X*, two homologous members of the neuroligin family, have been supposed as predisposition loci for autism spectrum disorders (ASDs), and defects of these two genes have been identified in a small fraction of individuals with ASDs. But no such rare variant in these two genes has as yet been adequately replicated in Chinese population and no common variant has been further investigated to be associated with ASDs.

**Methods:**

7 known ASDs-related rare variants in *NLGN3 *and *NLGN4X *genes were screened for replication of the initial findings and 12 intronic tagging single nucleotide polymorphisms (SNPs) were genotyped for case-control association analysis in a total of 229 ASDs cases and 184 control individuals in a Chinese Han cohort, using matrix-assisted laser desorption/ionization time-of-flight (MALDI-TOF) mass spectrometry.

**Results:**

We found that a common intronic variant, SNP rs4844285 in *NLGN3 *gene, and a specific 3-marker haplotype X^A^-X^G^-X^T ^(rs11795613-rs4844285-rs4844286) containing this individual SNP were associated with ASDs and showed a male bias, even after correction for multiple testing (SNP allele: P = 0.048, haplotype:P = 0.032). Simultaneously, none of these 7 known rare mutation of *NLGN3* and *NLGN4X* genes was identified, neither in our patients with ASDs nor controls, giving further evidence that these known rare variants might be not enriched in Chinese Han cohort.

**Conclusion:**

The present study provides initial evidence that a common variant in *NLGN3 *gene may play a role in the etiology of ASDs among affected males in Chinese Han population, and further supports the hypothesis that defect of synapse might involvement in the pathophysiology of ASDs.

## Background

Autism spectrum disorders (ASDs; MIM 209850) are acknowledged to be among the most heritable neurodevelopmental disorders with onset prior to age 3, which characterized by impaired reciprocal social interactions, deficient communication, restricted interests and stereotyped activity patterns (American Psychiatric Association, 2000). ASDs present a broadly defined disorders of behavior and cognition, including autism, Asperger syndrome and pervasive developmental disorder not otherwise specified (PPD-NOS)[1], which were conceptualized as a spectrum of disorders occurring on a continuum with a great diversity of symptom and varying degrees of severity. Asperger syndrome is diagnosed when lacks delays in cognitive development and language, and PDD-NOS is diagnosed when full criteria for the other two disorders are not met[2].

It is now well established that ASDs are etiologically heterogeneous, probably associated with a combination of the effects of genetic and environmental causes[3-5]. The strong role of genetic factors in the pathogenesis of ASDs has been definitely recognized, but still difficult completely comprehended. Approximately 10-20% of ASDs cases are already identified to be attributable to genetic syndromes with highly penetrant chromosomal abnormalities, known rare mutations, and copy number variants (CNVs) [1], but the causes of the majority of ASDs cases remain further determined and the biological basis remains pooly understood. Thus, neuroscience research efforts have focused on elucidating the neurobiological basis of ASDs, and a growing body of evidence indicates that ASDs are brain development disorders occuring during prenatal and postnatal period, and to put forward the possibility that the neuropathology of ASDs may present an on-going process extending through early childhood and into adulthood[6,7]. Moreover, histological studies on post-mortem brains have also revealed that neurons from autistic patients exhibit a reduction of GABA production, dendrite branching and arborization. Considering the fact that the normal functions of brain depend on proper working of the diverse synaptic components distributed in various compartments of the synapse, which is specialized site of cell-cell contact allowing communication between neurons, the synaptic hypothesis was proposed that abnormalities at the synapse may contribute to development of brain disorders such as ASDs and mental retardation. A genetics topic in ASDs has emerged focusing on identification of the synaptic genes contributing to the formation and function of the synapse. The synaptic hypothesis was also confirmed by the discovery of mutations in trans-synaptic adhesion proteins, such as neuroligins and neurexins, in a small fraction of individuals with ASDs, which was proposed to alter synaptic homeostasis and/or impair synapse development[8,9].

It is striking that ASDs show markedly skewed in sex distribution with approximately four times more frequent in male than female, together with the view that some of X-chromosomal genes may be involved in human neuropsychiatric disorders (X-linked disorders)[10], speculating that X chromosome may be of importance for the development of this condition. Many independent genome-wide scans for ASDs susceptibility loci have also demonstrated that X chromosome may contain more than one ASDs susceptibility genes. Two X-linked neuroligin genes, neuroligin 3 (*NLGN3*) and neuroligin 4X (*NLGN4X*) were therefore considered as good functional and positional candidate for predisposition to ASDs and have been screened for mutations in multiple studies.

Neuroligins are cell adhesion molecules localized postsynaptically in the glutamatergic synapses, and interact with presynaptic neurexins[11] to form heterophilic complex, which likely play an critical role in synaptic transmission and differentiation of synaptic contacts[12-14]. The role of neuroligins in ASDs was implicated in discovery deletions of Xp22.1 containing the *NLGN4X *gene in three autistic females[15]. Then Jamain et al.[16] first demonstrated the evidence for a pathogenic effect of neuroligins in ASDs, who identified a missense mutation (R451C) in *NLGN3 *and a frameshift mutation (1186insT) in the homologous gene *NLGN4X *in two Swedish families in each case one brother with typical autism and the other with Asperger Syndrome. The mutant proteins were considered significantly retained in the endoplasmic reticulum with reduced protein expression and/or adhesive activity to impair their function in the synaptogenesis compared with the wild-type neuroligin proteins[17,18]. Later, a 2-bp deletion (1253delAG) encoding a truncating *NLGN4X *protein (D429X) was detected in all individuals affected with mental retardation within a large French family[19], providing the evidence that rare mutations of these two neuroligin genes seem to contribute to developmental delay and ASDs. Animal model studies showed that mice carrying the R451C mutation of *NLGN3*[20] or a deletion of *NLGN4X *[21] caused an increase in inhibitory synaptic activity and exhibited behavioral phenotypes characteristic of ASDs due to protein misfolding [22], highlighting a possible role for excess inhibitory neurotransmission in ASDs. In additon, the specific *NLGN3 *mutation has also been considered to affect information processing in neuronal networks by altering network architecture and synchrony in a recent report [23]. Afterward, further genetic rare variants, including non-synonymous mutations and splice variants, in the *NLGN4X *gene were detected in probands with autism, mental retardation or pervasive developmental disorders-not otherwise specified (PDD-NOS) in following replication studies[24-27]. In contrast, controversial results obtained from several replication studies failing to detect neuroligin variants in samples of individuals with ASDs from different populations [28-31]. Moreover, no evidence was found for involvement of *NLGN3 *and *NLGN4X *with high-functioning ASDs in a 2008 study [32]. These results indicate that these rare mutations may be responsible for a small fraction of events and should been widely replicated and validated by other independent studies with different genetic background.

Meanwhile, there is emerging evidence indicating that the genetic etiology of complex traits of ASDs is likely to be based on a combination of multiple rare and common susceptibility loci. And common variants in susceptibility genes are also of importance for majority individuals, although exerting merely a modest or small effect sizes contributing to the genetic background of diseases[33]. However, little such variants in *NLGN3 *and *NLGN4X *gene have as yet been proven beyond doubt to be associated with ASDs [34]. So it is important to search for additional common variants for a better understanding of the genetic modulators of these two neuroligin genes responsible for ASDs susceptibility.

In the present study, we therefore attempted to replicate known rare variants in *NLGN3 *and *NLGN4X *genes to validate the initial findings and determine their frequencies in Chinese Han population, and further performed a case-control association analysis of tagging SNPs within these two neuroligin genes to evaluate if common variants also play a potentially role for etiology of ASDs.

## Methods

### Subjects

Subjects recruited in our study consisting of 229 unrelated patients with ASDs and 191 ethnically and geographically matched controls in Chinese Han cohort. All affected subjects (mean age: 5.5 years, 83.4% males, 16.6% females) were from outpatients of Children's Hospital, Zhejiang University School of Medicine and from several special autism-training schools, which were diagnosed using the diagnostic and statistical manual of mental disorders (DSM-IV) criteria or International Classification of Diseases-10 (ICD-10) by an experienced psychiatrist. In the present study, a relatively homogeneous sample of ASDs with typical phenotype was taken in to reduce heterogeneity. Clinical assessment and physical examination were obtained and the molecular tests were performed to exclude the patients with fragile X syndrome and tuberous sclerosis, or any kind of medical conditions suspected to be associated with ASDs. The controls (mean age: 3.2 years, 74.3% males, 25.7% females) sharing the same ethnical background with the affected subjects were unrelated Chinese individuals who had visited our hospital to evaluate their health status. They were clinically assessed to confirm the absence of both personal and family history of major neuropsychiatric disorders. The study was approved by Ethics Committee of Zhejiang University and written informed consents were obtained from parents or legal guardians for their participating in the study.

### Selection of rare mutations and SNPs

7 known rare variants in *NLGN3 *and *NLGN4X *genes previously reported to be associated with ASDs were chosen for rare variant screening (Table 1). The tagging SNPs were selected based on pair-wise r^2 ^method (parameter: r^2 ^≥ 0.8) using SNPbrowser software. Based on the genotype data from the SNP databases http://www.ncbi.nlm.nih.gov/snp/, 12 validated and suitable tagging SNPs within *NLGN3 *and *NLGN*4X genes (Table 2) with a minor allele frequency (MAF) greater than 5% in the Han_Chinese or in the HCB_Asian population were selected to capture the majority of the common variations for case-control association analysis.

**Table 1:** Summary and results of previously known rare variants in *NLGN3 *and *NLGN4X *gene

Gene	Sequence variant	Amino-acid change	Mutation type	Position in gene	Occurrence in ASDs/total ASDs**	Occurrence in controls/total controls**
***NLGN3***	c.C1351T	R451C	Missense mutation	Exon 6	0/229	0/188
***NLGN4X***	c.1186insT	D396X	Frameshift mutation	Exon 5	0/227	0/190
	c.1253delAG	D429X	Frameshift mutation	Exon 5	0/229	0/190
	c.759G > A	G99S	Missense mutation	Exon 3	0/229	0/191
	c.1597A > G	K378R	Missense mutation	Exon 6	0/227	0/191
	c.1671G > A	V403M	Missense mutation	Exon 6	0/229	0/191
	c.2574C > T	R704C	Missense mutation	Exon 7	0/229	0/189

**due to uncertainties, some sites were miss-identified for several samples, even by repeating.

**Table 2:** Allelic frequencies and case-control association analysis with ASDs in the primary and sex-specific analyses within the *NLGN3 *and *NLGN4X *genes

				Total samples (N = 420)					Male samples (N = 333)					Female samples (N = 87)				
				
Genes	SNP	Position in gene	Assoc Allele	Allele counts in controls(%)	Allele counts in cases(%)	P value	**P**_**corr **_**value**	OR (95%CI)	Allele counts in controls(%)	Allele counts in cases(%)	P value	**P**_**corr **_**value**	OR (95%CI)	Allele counts in controls(%)	Allele counts in cases(%)	P value	**P**_**corr **_**value**	OR (95%CI)
***NLGN3***	rs11795613 (G/A)	Intron1	X^A^	150(62.5%)	199(74.5%)	**0.0035**	**0.021**	**1.756 (1.201-2.567)**	84 (59.2%)	137(71.7%)	**0.0163**	ns	**1.752** **(1.106-2.774)**	66 (67.3%)	62(81.6%)	**0.035**	ns	**2.147** **(1.048-4.4)**
	rs4844285 (A/G)	Intron2	X^G^	149(62.6%)	201(75.6%)	**0.0016**	**0.0097**	**1.847 (1.259-2.711)**	83 (59.3%)	139(73.2%)	**0.0079**	**0.048**	**1.872** **(1.175-2.981)**	66 (67.3%)	62(81.6%)	**0.035**	ns	**2.147** **(1.048-4.4)**
	rs4844286 (G/T)	Intron2	X^T^	163(67.9%)	206(77.2%)	**0.0196**	ns	**1.595 (1.076-2.365)**	95 (66.9%)	145(75.9%)	0.07	ns	1.559 (0.963-2.525)	68 (69.4%)	61(80.3%)	0.104	ns	1.794 (0.882-3.648)
	rs5981079 (C/T)	Intron2	X^T^	132(55.5%)	175(67.0%)	**0.0079**	**0.047**	**1.634 (1.136-2.35)**	79 (55.6%)	126(66.7%)	**0.041**	ns	**1.595** **(1.019-2.497)**	53 (55.2%)	49(68.1%)	0.092	ns	1.728 (0.913-3.272)
	rs7051529 (G/A)	Intron4	X^A^	132(58.9%)	182(68.7%)	**0.025**	ns	**1.528 (1.054-2.217)**	73 (56.2%)	125(65.4%)	0.093	ns	1.479 (0.936-2.336)	59 (62.8%)	57(77.0%)	**0.047**	ns	**1.989** **(1.003-3.943)**
	rs10127395 (G/T)	Intron5	X^T^	196(81.7%)	221(83.1%)	0.676	ns	1.102 (0.698-1.743)	117(82.4%)	158(83.2%)	0.855	ns	1.055 (0.594-1.875)	79 (80.6%)	63(82.9%)	0.700	ns	1.166 (0.535-2.541)
***NLGN4X***	rs6529901 (G/A)	Intron3	X^A^	129(55.8%)	150(56.8%)	0.827	ns	1.04 (0.729-1.485)	82 (58.2%)	104(54.7%)	0.535	ns	0.87 (0.56-1.351)	47 (52.2%)	46(62.2%)	0.201	ns	1.503 (0.804-2.812)
	rs5961397 (G/A)	Intron3	X^A^	176(73.6%)	198(74.2%)	0.895	ns	1.027 (0.69-1.528)	106(75.2%)	134(70.2%)	0.312	ns	0.776 (0.475-1.27)	70 (71.4%)	64(84.2%)	**0.047**	ns	**2.133** **(1.001-4.545)**
	rs4370667 (C/T)	Intron2	X^T^	183(80.6%)	212(80.0%)	0.864	ns	0.962 (0.616-1.502)	112(81.8%)	150(79.4%)	0.592	ns	0.859 (0.491-1.501)	71 (78.9%)	62(81.6%)	0.665	ns	1.185 (0.549-2.559)
	rs10522049 (G/A)	Intron2	X^A^	198(82.8%)	223(83.5%)	0.839	ns	1.049 (0.658-1.35)	114(80.9%)	157(82.2%)	0.754	ns	1.094 (0.625-1.914)	84 (85.7%)	66(86.8%)	0.831	ns	1.1 (0.46-2.634)
	rs1882409 (A/G)	Intron1	X^A^	69(29.0%)	82(30.7%)	0.673	ns	1.086 (0.741-1.591)	42 (30.0%)	59(30.9%)	0.862	ns	1.043 (0.649-1.676)	27(27.6%)	23(30.3%)	0.695	ns	1.141 (0.590-2.208)
	rs5916352 (G/C)	Intron1	X^C^	184(77.0%)	215(81.7%)	0.187	ns	1.339 (0.867-2.067)	34 (24.1%)	32(17.1%)	0.117	ns	0.65 (0.378-1.117)	21 (21.4%)	16(21.1%)	0.952	ns	1.023 (0.492-2.128)

Assoc Allele, associated allele; OR, odds ratio; 95%CI, 95% confidence interval; P_corr _value, corrected by Bonferroni's approach for multiple testing, ns, not significant. Male samples (n = 333) include 191 cases and 142 controls, female samples (n = 87) include 38 cases and 49 controls. Significant P-values are indicated in bold.

### Rare Variants Screening and SNPs Genotyping

Genomic DNA was extracted from venous blood sample using AXYGEN DNA extraction kit (AxyPrep Blood Genomic DNA Miniprep Kit, USA), according to the manufacture's recommended protocol. Rare variant screening and SNPs genotyping were performed using matrix-assisted laser desorption/ionization time-of-flight (MALDI-TOF) mass spectrometry (Sequenom Inc., San Diego, California) and analyzed with the SpectroTYPER RT 2.0 software. A routine quality control procedure to assess genotyping reproducibility was performed by repeating 10% of samples selected randomly.

### Statistic analysis

Considering highly varying prevalence in female and male patients of ASDs, we separate subjects by gender into case and control subgroups. The Hardy-Weinberg equilibrium (HWE) for SNP markers on the X chromosome can be examined by testing two null hypotheses: H1: allele frequencies between males and females are equal and H2: HWE holds in females[35]. A basic association of allele frequencies was analyzed in PLINK version 1.07 software (http://pngu.mgh.harvard.edu/purcell/plink/; Purcell et al, 2009)[36] and corrected by Bonferroni's approach for multiple testing as the most stringent test to control false-positive results. The crude odds ratios (ORs) with 95% confidence intervals (95% CIs) were estimated for the effects of high-frequency allele in the case group. Pair-wise linkage disequilibrium (LD) between the different markers was estimated by the D' and r^2^, using Haploview version 4.2 software (http://www.broad.mit.edu/mpg/haploview Barrett et al., 2005)[37] with visualized structure. Haplotype blocks within each gene were defined by the default algorithm of Gabriel method[38] and haplotype frequencies were estimated with the expectation maximization (EM) algorithm, excluding haplotypes with frequencies lower than 5%. While for X-linked SNP in male samples, genotype of each SNP was interpreted as homozygous (genotype X^A^X^A ^or X^B^X^B^), although the male X was hemizygous (genotype X^A ^or X^B^). The exact P-values with 10,000 permutations were done for multiple test correction of the observed haplotype association. The differences were considered statistically significant with P < 0.05. Post-hoc power analysis was implemented using the computer G*Power.

SNPs were removed from analysis if they had the percentage of non-missing genotypes less than 0.95, displayed Hardy-Weinberg disequilibrium (P < 0.05), or had a minor allele frequency (MAF) < 0.05.

## Results

### Rare variants analysis

None of these 7 rare variants in *NLGN3* and *NLGN4X* genes previously known to associated with ASDs was replicated neither in our patients with ASDs nor controls (Table 1).

### Single nucleotide polymorphisms (SNPs) analysis

**Figure 1:**
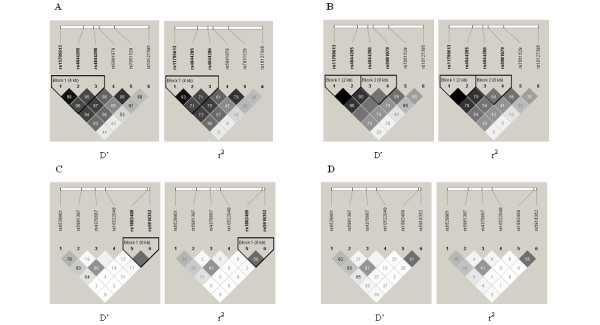
**LD of the SNPs of *NLGN3 *and *NLGN4X *genes**. The LD value (D' and r^2^) within each diamond was computed using Haploview. The dark gray diamonds indicate strong evidence of LD for no historical recombination, while the white diamonds indicate strong evidence of LD. The confidence bound values of the light gray diamonds are between the valves reflected in the dark and white diamonds. Haplotype blocks by the Gabriel method are shown also. A: LD of the SNPs of *NLGN3 *gene with D' and r^2 ^values for males, B: LD of the SNPs of *NLGN3 *gene with D' and r^2 ^values for females, C: LD of the SNPs of *NLGN4X *gene with D' and r^2 ^values for males, D: LD of the SNPs of *NLGN4X *gene with D' and r^2 ^values for females.

The genotypic distribution displayed no deviation from Hardy-Weinberg equilibrium (P > 0.05) (data not shown). Allelic frequencies and association findings of all these SNPs between patients with ASDs and controls in primary and sex-specific analyses have been summarized in Table 2. Pair-wise linkage disequilibrium (LD) between SNPs within each gene was calculated for males and females separately, and D' and r^2 ^values for all possible pairs were shown in Figure 1. Haplotype blocks generated by the Gabriel method were also shown in Figure 1. The results of the association analysis based on haplotype for each gene were summarized in Table 3.

**Table 3:** Haplotype blocks frequencies and case-control association analysis with ASDs within the *NLGN3 *and *NLGN4X *genes

Gene	Samples	Haplotype Sequence	Freqency in controls	Freqency in cases	Global P value	**χ**^**2**^	Individual P value	**P**_**per **_**value**	OR (95%CI)
***NLGN3***	Males	Block1: rs11795613-rs4844285-rs4844286							
		X^A^-X^G^-X^T^	0.579	0.716	**0.032**	6.74	**0.009**	**0.017**	**1.834(1.158-2.907)**
		X^G^-X^A^-X^G^	0.321	0.226		3.729	0.053	0.098	0.618(0.378-1.009)
		X^G^-X^A^-X^T^	0.079	0.042		1.975	0.160	0.462	0.515(0.202-1.317)
	Females	Block 1: rs11795613-rs4844285							
		X^A^-X^G^	0.673	0.816	**0.035**	4.458	**0.035**	0.08	**2.147(1.048-4.400)**
		X^G^-X^A^	0.327	0.184		4.458	**0.035**	0.08	**0.446(0.227-0.954)**
		Block 2: rs4844286-rs5981079							
		X^T^-X^T^	0.557	0.678	0.221	2.573	0.109	0.244	1.671(0.895-3.121)
		X^G^-X^C^	0.306	0.197		2.64	0.104	0.242	0.557(0.274-1.133)
		X^T^-X^C^	0.137	0.125		0.044	0.834	0.996	0.902(0.370-2.199)
***NLGN4X***	Males	Block 2: rs1882409-rs5916352							
		X^G^-X^C^	0.700	0.695	**0.035**	0.009	0.925	1	0.977(0.607-1.575)
		X^A^-X^G^	0.243	0.171		2.557	0.110	0.244	0.644(0.374-1.107)
		X^A^-X^C^	0.057	0.134		5.17	**0.023**	0.068	**2.546(1.112-5.832)**
	Females	Block 1**: rs6529901-rs5916397							
		X^A^-X^A^	0.514	0.595	0.145	1.122	0.290	0.665	1.386(0.756-2.539)
		X^G^-X^A^	0.200	0.247		0.557	0.456	0.831	1.315(0.641-2.697)
		X^G^-X^G^	0.269	0.129		5.083	**0.024**	0.060	**0.404(0.180-0.904)**

OR, odds ratio; 95%CI, 95% confidence interval. P_per _value, p value with 10,000 times permutation test. The haplotypes with frequencies lower than 5% excluded from analysis. Block 1**, the two SNP, rs6529901 and rs5916397, was in relatively high LD with D' > 0.8 and regarded as a block for multiple-marker analysis in this study. Significant p-values are indicated in bold.

As shown in Table 2, nominally significant differences of allele frequencies were detected for three SNPs in *NLGN3 *gene in total samples contrasted between individuals with ASDs and controls and these differences remained significant after Bonferroni correction for multiple testing. Giving the higher prevalence rate of ASDs in males than in females, we performed a secondary case-control analysis across all of SNPs stratified by sex. The sex-specific analysis showed that the significant differences of these three SNPs were almost entirely account for association with affected males. While only one SNP rs4844285 remained the evidence of significant association after Bonferroni correction testing (P_corr _= 0.048) and the rs4844285-X^G ^allele had an increased risk for male with ASDs (OR = 1.872, 95% CI:1.175-2.981). Pair-wise linkage disequilibrium (LD) revealed that the six SNPs analyzed were in high LD (D' > 0.8), and there were one three-marker haplotype block in male subgroup and two two-marker haplotype blocks in female subgroup generated by the default algorithm of Gabriel method (Figure 1). In male subgroup, a significant association was observed for the three-marker haplotype block (rs11795613-rs4844285-rs4844286, global P value = 0.032) containing the individual SNP rs4844285 associated with male ASDs in the allelic-wise analysis. The most frequent haplotype X^A^-X^G^-X^T ^was significantly more frequent in male patients with ASDs when compared to male controls (P = 0.009) and the diference remained after permutation testing (P_per _= 0.017), which suggested that the haplotype X^A^-X^G^-X^T ^play a role as a susceptibility factor for ASDs (OR = 1.834, 95% CI: 1.158-2.907). In female subgroup, one two-marker haplotype block (rs11795613-rs4844285) showed significant association with ASDs (global P value = 0.035). The haplotype X^A^-X^G ^was significantly more frequent in patients with ASDs compared with controls acting as a susceptibility factor for ASDs (P = 0.035, OR = 2.147, 95%CI: 1.048-4.400), while the haplotype X^G^-X^A ^was more frequent in controls likely acting as a protective factor against ASDs (P = 0.035, OR = 0.446, 95%CI: 0.227-0.954). These results did not survive 10,000 times permutation testing.

For *NLGN4X *gene, nominal significant difference in allelic frequency of SNP rs5916397 was detected between the female individuals with ASDs and controls (P = 0.047). The rs5916397-X^A ^allele was overrepresented in female individuals with ASDs (OR = 2.133, 95% CI = 1.001-4.545) while this difference disappeared for Bonferroni correction. Although no haplotype block containing this SNP was detected by the Gabriel method, this SNP was in relatively high LD with rs6529901 (D' > 0.8) and was available for the multimarker analysis. The result showed that no association was observed for this two-marker haplotype (rs6529901-rs5916397) both in males and females (males: global P value = 0.580, females: global P value = 0.145), even though the haplotype X^G^-X^G ^was significantly more frequent in controls compared to patients with ASDs in female subgroup (P = 0.024, OR = 0.404, 95%CI: 0.18-0.904). In addition, another two-marker haplotype block (rs1882409-rs5916352) was identified in male subgroup showing significant difference between patients with ASDs and controls (global P = 0.035), and haplotype X^A^-X^C ^was significantly more frequent in male ASDs compared to male controls (P = 0.023, OR = 2.546, 95%CI: 1.112-5.832), while the difference disappeared after permutation test.

### Power analysis

We estimated the statistical power using the G*Power program, based on the method of asymptotic noncentrality parameters for case-control association studies. Post-hoc power analysis showed that the used sample size had a 53.6% power to detect a small gene effect (corresponding to 0.1) and a 99.9% power to detect a medium gene effect (corresponding to 0.3) of the allele frequencies of SNP rs4844285 for a significant association at the alpha level of 0.05. Of the haplotype frequencies of rs11795613-rs4844285-rs4844286, the used sample size had a 37.5% power to detect a small gene effect (corresponding to 0.1) and a 99.9% power to detect a medium gene effect (corresponding to 0.3) at the alpha level of 0.05 (data not shown).

## Discussion

Our present results do lend support for the hypothesis that a common variant in the *NLGN3 *gene (SNP rs4844285) may influence the susceptibility for male ASDs. To our knowledge, this candidate gene study represents a focus association study of common variants of neuroligin genes in etioloty of ASDs, although previous studies have provide evidence of rare mutations of *NLGN3 *and *NLGN4X *genes causing to ASDs. Using a relative homogenous samples and a case-control based design, we found that an intronic SNP rs4844285 in *NLGN3 *gene and a specific 3-marker haplotype block (rs11795613-rs4844285-rs4844286) were associated with ASDs and showed a male bias after multiple correction, which was consistent with the fact that the prevalence rate of ASDs in males is higher than that in females. Simultaneously, further evidence was obtained for the fact that these known rare mutations in *NLGN3 *and *NLGN4X *genes may be not enriched in subjects with ASDs in Chinese Han cohort.

The neuroligin 3 gene (*NLGN3*) and neuroligin 4X gene (*NLGN4X*), localized on Xq13.1 and Xp22.33, encode a member of a family of neuronal cell surface proteins serving as splice site specific ligands for neurexins and may be involved in the formation, organization and remodeling of central nervous system synapses. The encoded proteins were localized postsynaptically and were shown to be sufficient to trigger the formation of new presynaptic architecture and induce the functional presynaptic differentiation in vitro[39]. So, it was speculated that genetic variants in these neuroligin genes may be associated with neurodevelopmental disorders, such as autism and Asperger syndrome, with a combination of rare variants having a dramatic effect in fewer individuals and common variants having small increments of risk in majority individuals. To date, only rare variants of these two neuroligin genes have been soundly established risk for ASDs responsible for a small fraction of events, while no common variant was available for susceptible to ASDs. For this reason, it is the most interesting finding in our present study that a common variant in the *NLGN3 *gene was identified to be strongly associated with susceptibility to male ASDs. The *NLGN3 *gene is composed of eight exons with the start codon in exon2[40] and the SNP rs4844285 implicated was an intronic common variant localizing in the intron 2 of the *NLGN3 *gene with unknown function. Our result showed that this SNP appeared to be most pronounced associated with an increased risk for ASDs among affected males after multiple testing corrections (P_corr _= 0.048), with rs4844285-X^G ^as risk allele (OR = 1.872, 95%CI:1.175-2.981). The finding was further supported by the multimarker analysis, which also showed, in male subjects, one three-marker haplotype block (rs11795613-rs4844185-rs4844286) containing this individual SNP was vulnerable to ASDs (global P value = 0.032) and the most frequent haplotype X^A^-X^G^-X^T ^was susceptible (P = 0.009, OR = 1.834, 95% CI: 1.158-2.907). However, how this specific intron relates to ASDs may be possible that this intronic SNP do not change gene function and a yet unidentified causal SNP may be linked to this haplotype and/or lies within or very close to the region spanning rs11795613 and 4844286, or a possibility of this intronic SNP playing pivotal roles in gene regulation. In addition, although nominally significant differences were detected for this SNP among female samples both in single SNP and haplotype analysis, the differences did not survive multiple testing corrections. The similar finding was obtained from another SNP rs5916397 of *NLGN4X *gene in female subjects, which showed nominal significant difference in allelic frequencies and haplotype X^G^-X^G ^(rs6529901-rs5916397) between the female ASDs and controls (allelic: p = 0.047, Haplotype X^G^-X^G^: p = 0.024), while this results disappeared for multiple correction. It was implicated that these SNPs may not make a statistically significant contribution to ASDs susceptibility in our female samples.

It was of particular meaningful that our result of association of SNP rs4844285 in *NLGN3 *gene with ASDs exhibited a male bias. Because of the overrepresentation of males in samples with ASDs, as well as evidence for sex-specific effects on X chromosome, we hypothesized that stratifying by gender may reduce genetic heterogeneity at these two X-linked neuroligin loci. One possible explanation of sex-specific association is that the underlying genetic modulators for ASDs may differ with gender. Indeed, a large body of researches have accumulated evidences that male and female brains develop differently not only in function field, but also that this sexual dichotomy extends to the macroscopic structures of the brain. Therefor, genes located on the X chromosome and those genes directly related to the function of sex-related hormones are clear candidates. There is also increasing evidence that differentially regulated genes on the autosomes are integrally involved in normal sex-specific brain development[41-43]. Evidences from multiple studies which have identified a major male-specific linkage peak at chromosome 17q11[44,45]. Although to what extent these regulatory effects may ultimately relate to normal male and female brain development remains unclear, it is obvious that there are substantial differences between the sexes at the molecular level. So, it is possible that *NLGN3 *may be causative gene for male predominance of ASDs. Another possible explanation is that the power to detect the observed difference reduces due to the small sample size of females in our present study. So, our sex-specific analyses were exploratory and require confirmation in additional samples.

Several notions of this study should be considered for this association. First, the tagging SNPs analyzed were unable to comprehensively cover the common variants across the both genes and other SNPs which were not selected in our present study may be associated with ASDs, further analysis on the basis of detailed SNPs coverage of these genes are necessary. In addition, all of the SNPs investigated in the present study exist in introns with unknown function and could not be regarded to be causally related to ASDs but rather is likely to be in linkage disequilibrium with an as yet untested, functional variant. Second, there were different haplotype blocks generated between the male and female group by the default algorithm of Gabriel method due to small difference of LD (D' and r^2 ^value) between pair-wise SNPs for different gender. Third, the Bonferroni correction was used for multimarker testing in our present study. It would be overly conservative for non-independent tests. Fourth, the average age of subjects was not well matched between the controls and ASDs in the present study. However, this should not be regarded to have a marked affect on the results, since genetic nature of SNP is generally unlikely to change with age.

Apart from this, rare genetic variants of susceptible genes are being increasingly discoveried as etiological factors in ASDs. But most instances, rare mutations of *NLGN3 *and *NLGN4X *genes have not yet been widely replicated and systemically validated by other independent studies within different population. Seeking replication in other ASDs samples is important to confirm the causative nature of these mutations and to determine their frequency. In our present study, we sought to replicate the 7 known rare variants associated with ASDs in both genes from Chinese Han population and revealed no mutations or rare variants. This lack of replication of rare variants of these two genes in our samples may be due to greater heterogeneity across different ethnic population, and highlights the fact that these known rare mutations may be not enriched in patients with ASDs in Chinese Han cohort. Moreover, it is important to note that, by current study design, sequencing analysis was not conducted and we could have missed other rare mutations potentially responsible for ASDs within these genes in our samples, such as those found in previous studies or likely to be de novo. Additional screening of much larger numbers of ASDs individuals from different ethnic backgrounds is needed to determine to what degree these genes are involved in the etiology of ASDs.

In summary, the present study provide initial evidence that common variants in *NLGN3 *gene may play a role in the etiology of ASDs among affected males in Chinese Han population, and further support the hypothesis that defect of synapse might involvement in the pathophysiology of ASDs. Our results of sex-specific association need replication and confirmation in independent samples. The molecular mechanisms of synaptic genes and X-linked genes remain excellent candidates for further genetic investigations responsible for etiology of ASDs.

## Competing interests

The authors declare that they have no competing interests.

## Authors' contributions

ZZ and JY designed and directed the entire study, HL, XH and DY are resposible for the inclusion of patients and controls, JY and ZL performed the experiments and stastistical analysis, JY wrote the manuscript.

All authors have read and approved the final manuscript.
